# Malignant Arrhythmia in Apical Ballooning Syndrome: Risk Factors and Outcomes

**Published:** 2008-08-01

**Authors:** Chadi Dib, Abhiram Prasad, Paul A Friedman, Elesber Ahmad, Charanjit S Rihal, Stephen C Hammill, Samuel J Asirvatham

**Affiliations:** 1 Division of Cardiovascular Diseases, Department of Internal Medicine, Mayo Clinic, Rochester, Minnesota; 2 Division of Cardiovascular Diseases, King's Daughters Medical Center, Ashland, Kentucky

**Keywords:** apical ballooning, Takotsubo cardiomyopathy, arrhythmia, sudden death, ventricular fibrillation, atrioventricular block

## Abstract

**Objectives:**

We sought to determine the frequency and outcomes with symptomatic arrhythmia in patients with apical ballooning syndrome (ABS).

**Methods:**

A retrospective review of the Mayo Clinic Angiography database was conducted to identify patients who met the Mayo criteria for ABS. Patients with documented arrhythmias formed the study group, and 31 randomly selected patients with ABS but without arrhythmia formed the control group.

**Results:**

Out of 105 patients identified with ABS, 6 (5.7%) women aged 69 +/- 9 years experienced significant arrhythmia (ventricular fibrillation, asystole), 2 patients died, and 1 required permanent pacemaker implantation. When compared with controls, the study group showed no significant difference with respect to  ECG  characteristics (QT, QRS duration or axis) except for  R-R  interval  variability (see comments below) (30.6±6 vs 14.5±17 p = 0.0004), QTc, and P-R interval. Patients without arrhythmia were more likely to be on  beta-blocker therapy than the study population (33% vs 80.6% p = 0.02).

**Conclusion:**

Life-threatening arrhythmia is uncommon (5.7%) with ABS despite marked, structural abnormalities. When arrhythmias do occur, the outcome is poor.  Prominent variability in R-R intervals appears to be predictive of significant arrhythmias in ABS.  The role of beta-blocker therapy in preventing arrhythmia with ABS requires further investigation.

## Introduction

Apical ballooning syndrome (ABS) also referred to as Takotsubo or stress cardiomyopathy is characterized by symptoms of myocardial ischemia, ST segment and T wave abnormality along with transient ventricular wall motion abnormalities in the absence of significant coronary artery stenosis [1,2]. There have been anecdotal reports for significant arrhythmias in patients with ABS [3,4], but the exact association, if any, of ABS with malignant arrhythmias is unknown. While ABS shares several similarities with acute coronary syndromes including QT interval prolongation and increased QT dispersion in the subacute phase [5], life-threatening arrhythmias and permanent conduction abnormalities appear to be less common.

Several studies comparing ABS with myocardial ischemia have found similarities in the ST segment and T wave changes including the time course of these changes in both sets of patients. An important distinction in the electrocardiographic findings between myocardial ischemia in ABS was recently reported [6]. Rate adaptation of ventricular repolarization, that is, the dynamicity of QT interval modulation in response to changes in heart rate, appears to be preserved in patients with ABS but is significantly affected in patients with myocardial infarction.

The aim of our study was to determine the incidence, significance, and outcomes of malignant brady- and tachyarrhythmia in patients with a definitive diagnosis of ABS. We further explored the potential contribution of the lack of effect of 24-hour R-R interval variability  on repolarization.

## Materials and Methods

### Study population

The Mayo Clinic Cardiac Catheterization database was retrospectively reviewed to identify patients who underwent coronary arteriography and left ventriculography and met the Mayo Clinic criteria (Table 1) [7] for diagnosis of ABS from January 1988 to November 2003. In addition, patients were enrolled prospectively from November 2003 to October 2006. We initially identified 550 patients without obstructive coronary disease and wall motion abnormality sparing the basal segments. Out of 550 patients, 421 were excluded because of a diagnosed cardiomyopathy, valvular disease, congenital heart disease, phaeochromocytoma, cocaine abuse, paced heart rhythm, or active myocarditis. Of the remaining 129 patients, 107 met the Mayo Clinic criteria for diagnosis of ABS [7] with documented complete normalization of left ventricular function on follow-up echocardiography, 2 patients could not be contacted, and the remaining 105 patients formed the study population.

### Data collection

The complete medical record with particular emphasis on historical features, medications, and 12-lead electrocardiograms were reviewed in the study population.

Initial echocardiography was performed without knowledge of patient outcome or eventual diagnosis.  Each echocardiogram of the patients in this series was reviewed by an independent echocardiologist who was blinded to patient outcome in this study. All diagnoses based on the echocardiographic features were found by both echocardiologists to be consistent with ABS.

### Electrocardiographic analysis

Manual measurement of the P wave duration, PR, QT, and R-R intervals were made. Bazett's formula was used to calculate the corrected QT interval[8]. QT variability was measured as the absolute difference in the QT interval in lead V1 from the QT interval in lead V6. This was done to understand the regional variation in repolarization given the regionality of wall motion abnormality in patients with ABS.  The PR interval was indexed to the heart rate to discount the potential effect of heart rate variation on AV nodal conduction. Each patient had a minimum of four electrocardiograms analyzed of which at least 1 was in the first 24 hours following diagnosis. Holter monitoring, telemetry, and other electrocardiographic data, when available, were analyzed. R-R interval variability was determined as the difference between the maximum and minimum heart rate during the 24 hours following diagnosis of ABS.

### Study and control group

Six patients with ABS were identified with life- threatening arrhythmia and formed the study group, and 31 patients were randomly selected from the remaining 99 patients without a fatal or life-threatening arrhythmia and formed the control group. The electrocardiographic parameter review and analysis was performed in a blinded fashion as to whether or not the patient had malignant arrhythmia.

The cause of death was confirmed using all available clinical data at the time of death and by reviewing the issued death certificates.  The study was approved by the Mayo Clinic Institutional Review Board for Human Research and included only patients who gave consent for their medical records to be utilized for research.

### Statistical analysis

Descriptive statistics are reported as frequencies and percentages for categorical data and the mean ± standard deviation for continuous data.  Comparisons between patient groups were performed with the Student t test for independent, categorical variables.  Follow-up events are reported as individual occurrences and estimated using the Kaplan-Meier method where applicable.

## Results

### Study group

Out of 105 patients with ABS (5.7%), 6 were found to have life- threatening arrhythmias and formed the study group.  All patients were females with mean age of 69 ± 8.9 years (Table 2). Salient features include the average heart rate at presentation 89 ± 26.8 bpm, P wave duration 100 ± 20 ms, PR interval 207 ± 96 ms, PR index 2.1 ± 1.4 ms/beat, QT interval 405 ± 85 ms, QTc 491 ± 81 ms, QT dispersion 78 ± 40.2 ms, regional QT variability (QT in lead V6 - QT in lead V1) 11 ± 23.4, and maximal 24-hour R-R interval variation 30.6 ± 6 ms (Table 3). The median and interquartile range from time from onset of symptoms to ECG was 100 and 12.5-338.5 minutes.

Out of the 6 patients who presented with ventricular fibrillation, 2 were first diagnosed with  ABS following resuscitation. *Patient 1* had sinus pauses at presentation with ABS up to 7.4 seconds in duration. The clinical course was later complicated by asystole requiring resuscitation that included temporary pacemaker implantation and inotropic support with gradual clinical improvement.  *Patient 2* had a documented cardiac arrest 4 days after diagnosis with ABS. The QTc was 399 ms 3 hours prior to her arrest. The patient did have a prior history of atrial fibrillation but was not monitored electrocardiographically at the time of cardiac arrest. Ventricular fibrillation was documented during attempted resuscitation that failed with the patient dying. *Patient 3* had a prior history of multifocal atrial tachycardia and pacemaker implantation and presented with ventricular fibrillation. The patient recovered completely and was discharged home uneventfully 9 days following diagnosis but died following sudden cardiac arrest 3 days following hospital discharge. *Patient 4* presented with unexplained collapse and documented ventricular fibrillation. Following resuscitation, a diagnosis of ABS was made. The patient gradually improved and was discharged with subsequent complete normalization echocardiographically and clinically at 7 months post episodes. *Patient 5* presented with anginal chest pain, and admission  ECG  showed Mobitz I AV block and right bundle-branch block at the time of diagnosis with ABS.  One week following hospitalization, worsening of Mobitz I block along with evidence of sinus node dysfunction was found. EP study showed no evidence of infra-Hisian conduction disease. The patient had completely recovered but with persisting right bundle-branch block and Mobitz I AV block 16 months following ABS diagnosis. *Patient 6* had electrocardiographically documented Mobitz II AV block with ventricular rate of 32 bpm. Permanent pacemaker implantation was performed, and the remainder of her hospital course was uneventful with complete recovery of wall motion abnormalities at follow-up up to 7 years following initial diagnosis.

### Control group

Thirty-one patients with diagnosed ABS without significant arrhythmia constituted the control group. All patients were female with mean age of 68.2 +/- 12 years, average heart rate of 86.5 +/- 21 bpm, PR interval of 162 +/- 24 ms, QT interval of 366.4 +/- 52 ms, QTc interval of 433.7 +/- 25 ms, and maximal 24-hour R-R interval *variation* of 14.5 +/- 17 ms. The median and interquartile range from time from onset of symptoms to ECG was 240 and 5-870 minutes.

### Differences in study and control groups

**Figure 1:**
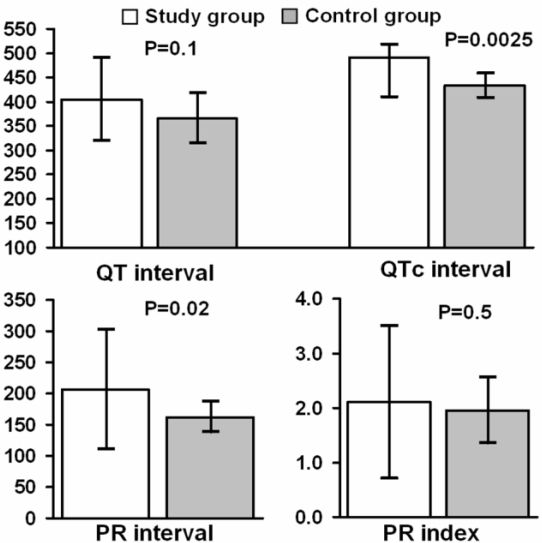
*Upper panel:*  There was no significant difference in the baseline QT interval between patients with ABS with and without malignant arrhythmia.  However, the corrected QT interval was significantly different, being higher in the patients with arrhythmias (see text for details). *Lower panel:*  There was a significant difference in the PR interval in the study versus control group. The PR interval was longer in patients with arrhythmia and ABS. When the PR interval was indexed for the heart rate, there was no difference in the two groups. This suggests that at higher sinus rates, the PR interval shortened in ABS patients with arrhythmias. This in turn suggests that the cause of AV delay was at the level of the compact AV node, and conduction improved during catecholamine stress (see text for details).

In comparing the patients with and without malignant arrhythmia, there was no statistically significant difference in the heart rate at presentation (89±26.8 vs 86.5±21.2, p = 0.8), P wave axis (73.3±13.3 vs 57.6±17.7, p = 0.1), QT interval (405.3±84.7 vs 366.4±52.3, p = 0.1), QT index (4.55±6.39 vs 5.09±10.41, p = 0.9), QT dispersion (78±40.2 vs 63.3±26.7, p = 0.2), regional QT variability (11±23.4 vs 15.5±34.1, p = 0.8), PR index ( 2.1±1.4 vs 1.95±0.6, p = 0.5), or time of onset of symptoms to ECG (median 100 vs 240, p = 0.2) (Figure 1).

**Figure 2:**
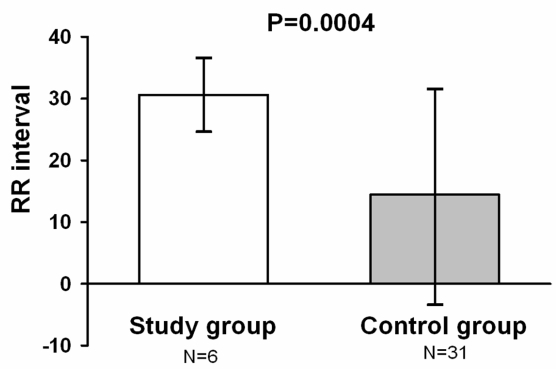
The maximal R-R interval variation at first evaluation was significantly greater in patients who developed life-threatening arrhythmia when compared to the control group with ABS and no significant arrhythmia. This finding, coupled with our observation of longer corrected QT interval in the study group suggests potential mechanism for arrhythmogenicity in ABS patients who developed documented malignant arrhythmia.

There was a statistically significant difference between these 2 groups in maximal R-R interval variation (30.6±6 vs 14.5±17, p = 0.0004), QTc interval (490.5±80.8 vs 433.7±25.4, p = 0.0025), and PR interval (207±95.9 vs 162±24.3, p = 0.02) (Figure 2).

Clinical characteristics at baseline were similar in the control and study groups (age, sex, hypertension, diabetes). Patients in the study group, however, had a higher prevalence of atrial fibrillation (66.6% vs 9.6% p = 0.001) and were less likely to be on beta-blocker therapy at the time of presentation (2/6 [33.3%] vs. 25/31 [80.6%] p = 0.02).

## Discussion

Since its recent description as a clinical entity characterized by transient ventricular dysfunction in the setting of chest pain, ST segment, and T wave abnormalities in the absence of occlusive coronary artery disease, apical ballooning syndrome has become the subject of significant investigation [1,2,5,6]. While important advances have been made in understanding the extent of myocardial involvement [1,2], potential pathogenic mechanisms, and distinctions with ischemic syndromes [1,3,5,6], little is known about the risk and outcomes associated with arrhythmia (bradycardic and tachycardic) occurring in the setting of ABS.

Previous reports have shown that although the type of changes in the 12-lead surface electrocardiogram and the time course of these ECG  changes in ABS are similar to those seen in patients with a myocardial infarction [5,9],  malignant arrhythmias are much less common in ABS. Matsuoka et al [5] reported the absence of sudden cardiac death and other malignant ventricular arrhythmia in patients with ABS even though electrocardiographic abnormality including significantly prolonged QT intervals and QT dispersion persisted for several weeks after the index event. A few prior reports have, however, demonstrated occasional apparent association between ABS and both significant bradyarrhythmia and tachycardia [3,4].

In our study population of 105 patients diagnosed with ABS based on Mayo Clinic criteria, 6 patients (5.7%) experienced life-threatening arrhythmia and/or conduction abnormality. There was no significant difference in these patients with malignant arrhythmia compared to a control population (ABS without arrhythmia) with regard to clinical presentation, age, sex and other clinical parameters. We did, however, find several important electrocardiographic and previous history differences that may provide clues to the pathophysiology of ABS and point to treatment options to decrease risks of these arrhythmias.

### Maximal RR interval variation

**Figure 3:**
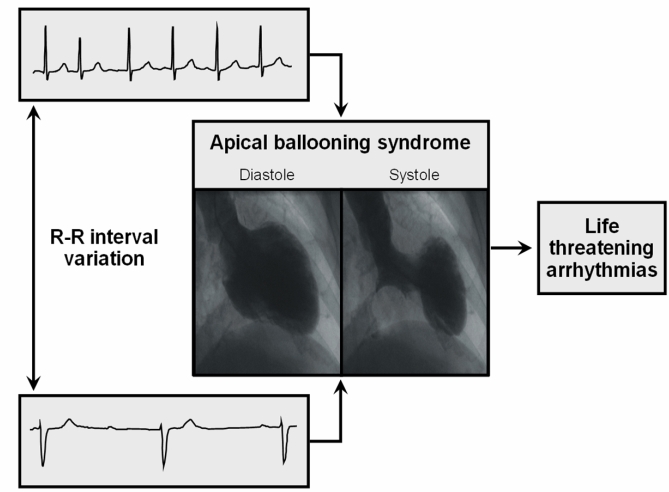
All patients in the study and control group met our published criteria for ABS.  Although all patients had the typical transient ST segment and T wave changes at the time of documented wall motion abnormality, patients who developed malignant arrhythmia had greater variation in the R-R intervals along with a longer corrected QT interval (see text for details).

We report that patients with ABS who had malignant arrhythmia had significantly more variation in their R-R intervals (maximal R-R interval - minimum R-R interval in the first 24 hours of admission) compared with the control group. A recent report by Bonnemeier et al [6] may help explain our findings. In their study [6], patients with ABS continued to show modulation of the QT interval with changes in the heart rate even during the acute phase of the syndrome. This was in contradistinction to the absence of such modulation in patients with myocardial infarction [10]. Thus, when malignant arrhythmias occur in myocardial infarction, they are likely as a result of ischemia itself, whereas in apical ballooning syndrome should a patient have prior QT prolongation or have significant heart rate variation, they would likely have more prominent QT interval changes that may increase the propensity for malignant arrhythmia (Figure 3).

### Prior arrhythmia

We found a significant increase in prior arrhythmia in patients with ABS who developed malignant arrhythmia. Three patients had prior atrial fibrillation, and 1 patient each with multifocal atrial tachycardia, variable AV node dysfunction, and sinus node dysfunction. Each of these conditions increases the variability of ventricular activation (variable heart rate). As discussed above, with the recently demonstrated continued presence of QT modulation with heart rate variation, these prior arrhythmias may have contributed to the study group's predilection for malignant arrhythmia.

### PR and QT intervals

In our study, we found a statistically significant difference in the PR intervals between the 2 groups, yet there was no difference once we corrected for heart rate (PR index).  Thus, the patients with the longer PR intervals had concomitant slower heart rates.  This finding suggests that the PR prolongation is not from intrinsic AV nodal conduction disease but from sympathovagal imbalance as a previously suggested abnormality in ABS [11]. Structural AV nodal disease as potentially from transient disturbances in the microcirculation as previously suggested [4] would have resulted in longer PR intervals at *faster* heart rates and is contrary to our finding. We further found evidence in our 2 patients with AV block that support a predominant catecholamine surge is important in the pathogenesis of ABS. In the patient with Mobitz I AV block and EP study proven absence of intra-Hisian conduction disease, there appeared to be transient improvement in AV conduction in the acute phase of the syndrome. On the other hand, in the patient with Mobitz II AV block (infra-Hisian), the condition was worsened in the acute phase. This type of response in AV block is consistent with catecholamine stimulation [12].

We also found in our study that the QT interval corrected for heart rate (QTc) was significantly longer in patients at risk for malignant arrhythmia even though the absolute QT interval showed no difference.  This finding suggests that in our study group, the longer QT interval occurred in patients with relatively faster heart rates, a finding similar to that observed in ischemic heart disease.  As discussed previously, because of the preserved QT modulatory defects of heart rates in ABS [6] (as opposed to its absence in ischemic heart disease), this may have further contributed to arrhythmogenesis seen in our patients with marked rate variability. A second explanation may be that because the malignant arrhythmias occurred in our patients with marked variation in their heart rate, the standard correction for QT intervals at extremes of heart rate may have been inaccurate. It is possible that in a few patients who had cardiac arrest or other severe medical illness/dyselectrolytemia that the QTc at the time of the measurement used in our study may have been affected. Whenever possible, pre-event (cardiac arrest) electrograms were used to obtain baseline data.

### Beta-blocker use

An unanticipated finding in our study was the statistically significant difference in beta-blocker use in the study group 2/6 (33.3%) versus the control group 25/31 (80.6%), p value 0.02.  While this result should be interpreted with caution given the relatively small number of patients in the study group, it nevertheless is consistent with the hypothesis that marked variation in heart rate may be the primary factor increasing the risk for malignant arrhythmia in patients with ABS.  If further studies also show such a protective effect of beta-blockers, then recommending these drugs for long-term use following recovery may be considered given the nonnegligible recurrence rate for ABS (11.4% at 4-year follow-up1).

Another incidental finding of potential significance from our study was the higher prevalence of atrial fibrillation (66% vs. 9.6%, p=0.001) in the study group with malignant arrhythmia compared to the control group. Based on the above discussion on preserved QT dynamicity in ABS patients, the reason for this increased prevalence of atrial fibrillation may be as a result of prominent R-R interval variability from this disease, in turn producing significant ventricular arrhythmia. Alternative possibilities include preexisting conduction abnormalities in patients with atrial fibrillation (sick sinus syndrome) or a previously unproven association of atrial fibrillation with ABS.

Thus, while our study shows that malignant arrhythmias are uncommon in patients with ABS, they do occur, and marked heart rate variability and previously diagnosed arrhythmia may be risk factors for their development while beta-blocker use may be protective. Our study was too small to comment on ICD benefit. However, in one patient who had a resuscitated event, secondary prophylaxis could have been considered and may have prevented his event.

## Limitations

A portion of our study was conducted in a retrospective fashion, and thus some data relevant to arrhythmias may have not been collected at the time of diagnosis. We did perform, however, a careful chart review and all saved electrocardiographic and rhythm monitoring data to try and offset this weakness in our study.

We used the simple difference between maximal and minimal R-R intervals during 24-hours to assess for R-R interval variability in our study. A more robust method of looking at heart rate variability would have been superior given the importance of this finding in our study. However, similar measurements in both the control and study group were made in a blinded fashion, thus the reported association is likely accurate.

Another limitation of our study is that we have grouped arrhythmias of varying pathophysiology as malignant arrhythmias and defined our study group accordingly. The majority of the patients did have ventricular fibrillation, but as noted in our discussion, AV nodal block may accentuate the propensity for ventricular arrhythmia as well.  In addition, our findings that prior beta-blocker use was protective and that marked R-R interval variability was predictive of arrhythmic events regardless of whether these were tachyarrhythmic or bradyarrhythmic constituted the rationale for this grouping.

In our study, we excluded many patients (421/550 [76.5%]) with likely ABS when a concomitant cardiac diagnosis (dilated cardiomyopathy, etc.) was present. In practice, patients with these previous comorbidities may develop ABS, and the risk factors for malignant arrhythmia and possible protective effect of beta-blockade may be different from our findings.

## Conclusion

To our knowledge, this is the first report in a relatively large series of confirmed ABS patients on the occurrence of malignant arrhythmias specifically comparing patients with such arrhythmias with control patients with ABS but without clinical arrhythmia.  We found that marked 24-hour R-R interval variation and prior history of arrhythmia increased the propensity for malignant arrhythmia in patients with ABS, and beta-blocker use decreases the risk of such arrhythmia.

If future, larger studies confirm these observations, then more careful monitoring of patients with ABS, prior arrhythmia, and marked heart rate variation is warranted, and beta-blocker use can be recommended particularly in anticipation of possible recurrent ABS.

## Figures and Tables

**Table 1:**
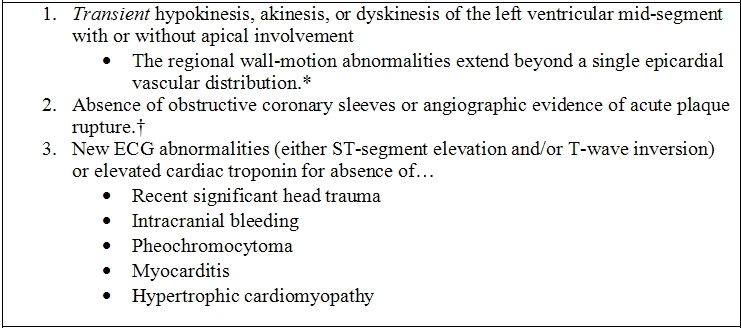
Mayo Clinic Criteria for the Clinical Diagnosis of Apical Ballooning Syndrome

* Rare exceptions to these criteria exist. † The possibility of ABS developing along with obstructive coronary atherosclerosis may rarely need consideration. In either of the above circumstances, the diagnosis of apical ballooning syndrome should be made with caution, and evidence for a clear stressful precipitating figure must be sought.

**Table 2:**
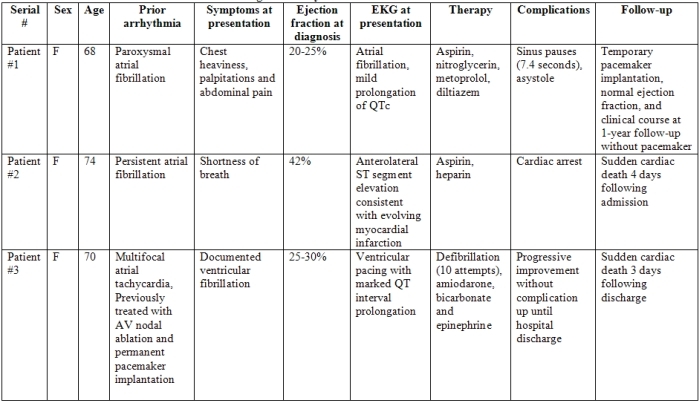

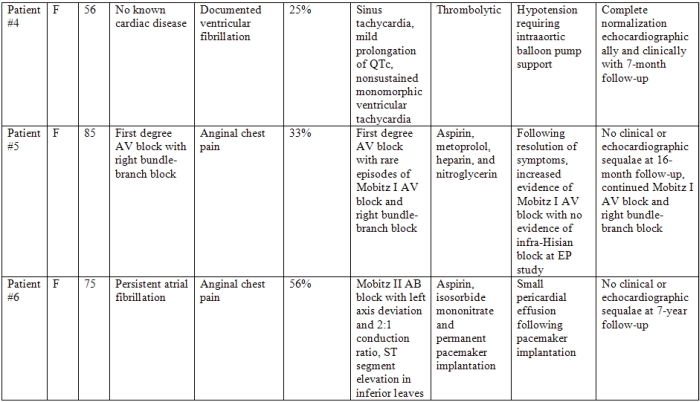
ABS and Malignant Arrhythmia: Patient Characteristics

**Table 3:**
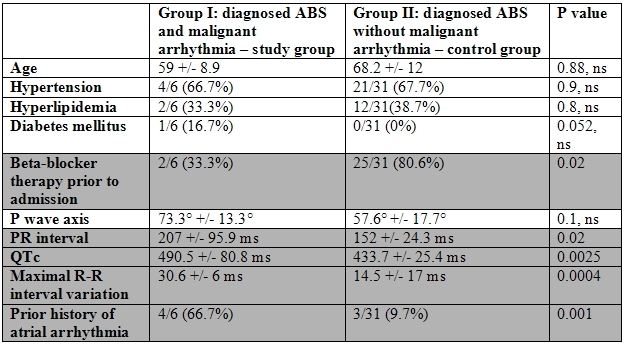
Malignant Arrhythmia and ABS: Risk Factors
